# Construction of a Conceptual Framework for Assessment of Health-Related Quality of Life in Dogs With Osteoarthritis

**DOI:** 10.3389/fvets.2021.741864

**Published:** 2021-09-24

**Authors:** Claire Roberts, Bryony Armson, David Bartram, Zoe Belshaw, Hannah Capon, Georgina Cherry, Laura Gonzalez Villeta, Shona L. McIntyre, Isaac Odeyemi, Alasdair J. C. Cook

**Affiliations:** ^1^vHive, School of Veterinary Medicine, University of Surrey, Guildford, United Kingdom; ^2^Outcomes Research, Zoetis, Loughlinstown, County Dublin, Ireland; ^3^EviVet Research Consultancy, Nottingham, United Kingdom; ^4^Canine Arthritis Management, Shoreham-by-Sea, United Kingdom

**Keywords:** osteoarthritis, conceptual framework, quality of life, degenerative joint disease, dog, pain, canine

## Abstract

An owner's ability to detect changes in the behavior of a dog afflicted with osteoarthritis (OA) may be a barrier to presentation, clinical diagnosis and initiation of treatment. Management of OA also relies upon an owner's ability to accurately monitor improvement following a trial period of pain relief. The changes in behavior that are associated with the onset and relief of pain from OA can be assessed to determine the dog's health-related quality of life (HRQOL). HRQOL assessments are widely used in human medicine and if developed correctly can be used in the monitoring of disease and in clinical trials. This study followed established guidelines to construct a conceptual framework of indicators of HRQOL in dogs with OA. This generated items that can be used to develop a HRQOL assessment tool specific to dogs with OA. A systematic review was conducted using Web of Science, PubMed and Scopus with search terms related to indicators of HRQOL in dogs with osteoarthritis. Eligibility and quality assessment criteria were applied. Data were extracted from eligible studies using a comprehensive data charting table. Resulting domains and items were assessed at a half-day workshop attended by experts in canine osteoarthritis and quality of life. Domains and their interactions were finalized and a visual representation of the conceptual framework was produced. A total of 1,264 unique articles were generated in the database searches and assessed for inclusion. Of these, 21 progressed to data extraction. After combining synonyms, 47 unique items were categorized across six domains. Review of the six domains by the expert panel resulted in their reduction to four: physical appearance, capability, behavior, and mood. All four categories were deemed to be influenced by pain from osteoarthritis. Capability, mood, and behavior were all hypothesized to impact on each other while physical appearance was impacted by, but did not impact upon, the other domains. The framework has potential application to inform the development of valid and reliable instruments to operationalize measurement of HRQOL in canine OA for use in general veterinary practice to guide OA management decisions and in clinical studies to evaluate treatment outcomes.

## Introduction

Osteoarthritis (OA), also known as degenerative joint disease, refers to the irreversible degeneration of cartilage and other tissues within joints. Prevalence estimates in the late 1990s have been as high as 20–30% of dogs over the age of 1 year (1, 2). A recent study using a canine OA screening checklist indicated that 37% of screened dogs had clinical signs associated with OA (3). OA can occur in any dog although it is more often diagnosed in older dogs and dogs of certain large breeds (4). Pain associated with OA has a broad impact in dogs through its effects on gait and movement, function, affective state, social relationships and sleep (5). Foundational to OA treatment is the proactive management of pain over the course of the disease to help ensure the success of other supportive care (1). UK veterinarians rate OA to be the most common cause of pain in dogs (6).

Owners may not recognize signs of OA that are presented by their dogs and this can be a major barrier to initiation of treatment (7). Case management often involves a trial period of pain relief treatment (7), the evaluation of which relies on owners monitoring changes accurately and noticing any improvements to quality of life (QOL). Quality of life related to a disease, or health-related quality of life (HRQOL), in dogs can be defined as “in the context of an altered health state and associated health interventions, the evaluation by the individual of its circumstances (internal and external), and the affective (emotional) response to those circumstances” (8). Humans with osteoarthritis have reported that it causes significantly lower levels of quality of life (9–13). It is likely that OA affects numerous aspects of quality of life in dogs (5, 14–16).

Humans are generally able to assess their own quality of life. This may be performed using a patient-reported outcome measure (PROM), an instrument that allows the patient to report on aspects of their own health, such as pain and QOL (17, 18). Non-human animals are unable to report their own health outcome and require a proxy, which for dogs is generally the owner. Generic and disease specific HRQOL measures have been developed for dogs (19–22). The use of both approaches has been reported in veterinary literature (23). Disease-specific measures of QOL have the advantage of frequently being more responsive (sensitivity to detect change over time) and clinically useful than generic QOL measures which do not focus on any specific condition (24) while generic measures have the advantage of enabling comparisons of QOL burden and treatment benefit across diseases (25).

HRQOL assessments are widely used in human medicine. The Food and Drug Administration (FDA) patient-reported outcome (PRO) guidance outlines methods for the development and validation of human PROs and HRQOL measures that can be used to support claims in medical product labelling (26). This guidance recommends the initial development of a conceptual framework (CF), which is a model that identifies the QOL domains that are affected by the health-related condition of interest and the relationship between them. This paper presents a CF for HRQOL in dogs with OA. The CF will be used to generate initial items for a PROM instrument. Conceptual frameworks have already been used to inform the development and comprehensiveness of companion animal QOL measures (27).

The current study aimed to construct a conceptual framework of indicators of HRQOL in dogs with OA, focusing on the subjective experience of the dog. The CF was based upon a systematic literature review that was reviewed by an expert panel. The framework has potential application to inform the development of valid and reliable instruments to operationalize measurement of HRQOL in canine OA for use in general veterinary practice to guide OA management decisions and in clinical studies to evaluate treatment outcomes.

## Materials and Methods

### Literature Search

**Figure 1:**
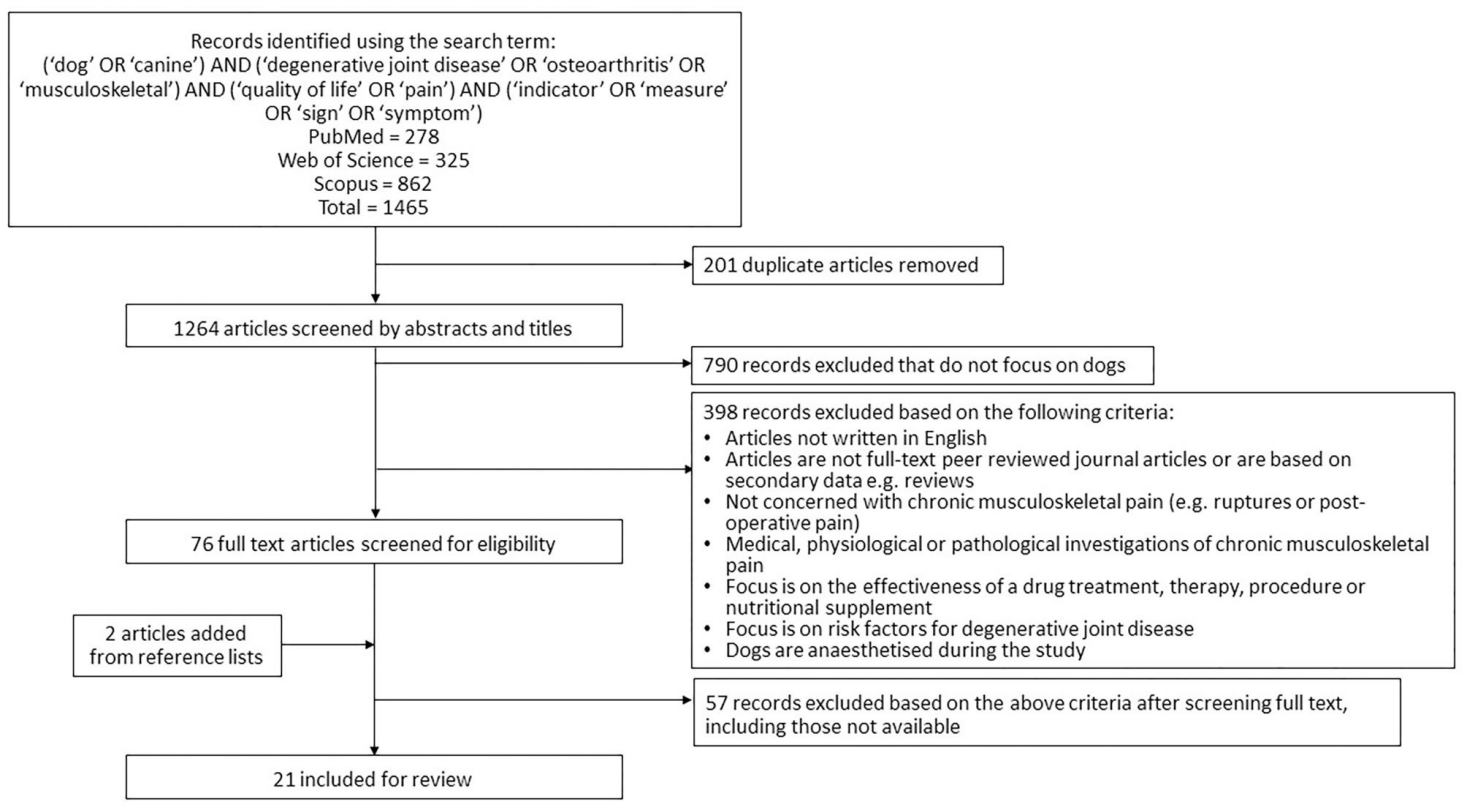
Flow diagram summarizing the literature search on canine osteoarthritis and application of eligibility criteria.

A systematic literature review was performed in August 2020 following the Preferred Reporting Items for Systematic Reviews and Meta-analyses (PRISMA) guidelines (28). Three scientific databases (Web of Science, PubMed and Scopus) were searched with the terms [(“dog” OR “canine”) AND (“degenerative joint disease” OR “osteoarthritis” OR “musculoskeletal”) AND (“quality of life” OR “pain”) AND (“indicator” OR “measure” OR “sign” OR “symptom”)]. Duplicate articles were removed. Remaining studies were assessed for inclusion by multiple authors (BA, GC, SLM, LGV) using the following predefined eligibility criteria: (i) focused on dogs (ii) published in or after the year 2000, (iii) available in the English language, (iv) peer reviewed (v) original research and (vi) focused on indicators of chronic musculoskeletal pain or its impact on quality of life. Studies concerning acute pain, risk factors for pain or on treatment effectiveness were excluded (Figure 1). Studies were initially assessed by screening of the title and the abstract; remaining articles were assessed by reading the full article. The bibliographies of eligible search result articles were checked for further additional eligible articles.

Quality assessment was performed using a modified version of the STROBE (Strengthening The Reporting of Observational studies in Epidemiology) guidelines (29). This consisted of twelve criteria (Supplementary Table 1). Articles meeting six or more of the criteria were eligible for progression to data extraction. Bias of risk within studies was addressed within the quality assessment.

### Data Extraction

Indicators of osteoarthritis and/or its impact on quality of life were extracted to generate “items” from each study. Only indicators listed within the body of the manuscript were included. Data were initially extracted by one of four authors (BA, GC, SLM, LGV), with the process repeated independently by a single author (CR) for all studies. A comprehensive data charting table based on a previous review for HRQOL in cats with osteoarthritis (30) and refined iteratively was used to extract relevant information from included sources of evidence. This consisted of nine domains: mobility, physical appearance, energy/vitality/behavior, temperament, pain expressions, sociability and well-being. Items were cross checked between studies to identify duplications, and the frequencies of each item were recorded. Items that were deemed to be synonyms (e.g., “weary” and “tired”) were combined by the authors as applicable.

### Expert Workshop

The resulting domains and items were assessed at a workshop attended by members of the research team, a specialist in small animal internal medicine with a PhD in decision making in dogs with osteoarthritis and a veterinary surgeon who founded an initiative to provide advice and education on canine arthritis to owners and veterinary professionals. Prior to the meeting, panel attendees were provided with a PowerPoint file covering the methodology and results including a table of key domains/items (Supplementary Table 2) and a brief for the meeting. The meeting brief was to assess the appropriateness of nomenclature for domains, whether each of the items were in the “correct” domain and whether any of the items could be combined (i.e., synonymous). The brief also stated the aim of the meeting which was to establish a hypothesis for how the domains interrelate and to prepare a diagram to illustrate the domains and their interactions.

The workshop took place in November 2020 online and lasted 2 1/2 h. Domains were discussed and final titles decided, with each item assessed for designation to the correct domain. Additionally, any potential missing items were discussed. Hypothesized directional interactions between the domains were identified and a visual representation of the conceptual framework was produced.

## Results

A total of 1,264 unique articles were generated in the database searches and assessed for inclusion using the title and abstract (Figure 1). Of these, 76 progressed to have the full manuscript screened. Two articles were added from reference lists (14, 31). Twenty-one studies were identified to be eligible for inclusion in the data extraction process (8, 14–16, 31–48). Quality assessment allowed all 21 to progress (Supplementary Table 1).

The eligible studies dated from 2003 to August 2020 inclusive. Study characteristics are shown in Supplementary Table 3. All studies bar one (45) addressed the risk of bias. Sixteen (76.2%) of the studies described the development (*n* = 11), translation (*n* = 2), or use (*n* = 3) of pain or HRQOL assessment instrument[s]. There were five instruments: the Liverpool Osteoarthritis in Dogs (LOAD) score, (31, 46), Canine Brief Pain Inventory (CBPI), (15, 38, 39, 44, 46), Helsinki Chronic Pain Index (HCPI) (16, 38, 41, 42, 46), the Canine Orthopedic Index (COI) (32, 33, 36, 37) and the Glasgow University health-related dog behavior questionnaire (GUVQuest) (8, 45, 48).

Data extraction produced 134 unique items (Supplementary Table 2) categorized across domains as follows: physical appearance six items (4.5%); mobility 28 items (20.9%); energy/vitality/behavior 35 items (26.1%); temperament 47 items (35.1%); pain expression 11 items (8.2%); sociability 7 items (5.2%). The domain “well-being” was removed from the list of domains as all items within well-being could also be categorized within one of the other domains. The most common items (in six or more studies) were: rising from lying, climbing up, vocalization, climbing down, jumping up, jumping down and general activity. Over half of the items were unique to a single study (77/134; 57.5%). Items ranged from 0–59 per study, with an average of 12.5 items per study. Combining synonyms reduced the number of items to 47.

The expert workshop resulted in four remaining categories: physical appearance, capability, behavior, and mood (Table 1). The original domain “physical appearance” was retained as a heading. “Mobility” was renamed “capability” to reflect the ability of a dog to perform certain tasks not necessarily related to moving, such as staying upright in a car. The domain “energy, vitality and behavior” was shortened to “behavior” and was thought to incorporate all the items previously within the “sociability” domain, such as attention seeking. “Temperament” was renamed “mood” after the top item within the domain and to reflect that the items in the domain can be either positive or negative. The items within the domain “pain expressions” were incorporated into other domains, as all items were deemed to be reactions to pain.

**Table 1:** Domains and items resulting from a literature review on indicators of HRQOL in dogs with osteoarthritis, after categorization by an expert panel.

**Domains**			
**Physical appearance**	**Capability**	**Behavior**	**Mood**
“Sad” appearance of eyes	Willingness to walk	Reluctance to move/exercise	Low mood
Trembling/shaking leg	Willingness to trot	Slower during exercise	Uninterested
Excessive panting	Willingness to gallop	More resting time during walks	Apprehensive
Awkward posture	Ability to jump	Sleeping more	Lethargic
	Ability to lie down	Less time playing	Frightened
	Ability to rise from rest	Change in appetite	Detached
	Ability to climb up (e.g., stairs)	Not pulling on lead	Quiet
	Ability to climb down (e.g., stairs)	More sniffing on walks	Unresponsive
	Stay upright in moving car	Vocalisation	Withdrawn
	Posture to toilet	Attention seeking	Aggressive
	Gait alteration	Comfort seeking	Irritable
	Limping during and after activity	Unsociable	Protective
	Lameness after rest		Depressed
	Stiffness during and after activity		Confused
	Stiffness after rest		Less confidence
	Severity of limp		

**Figure 2:**
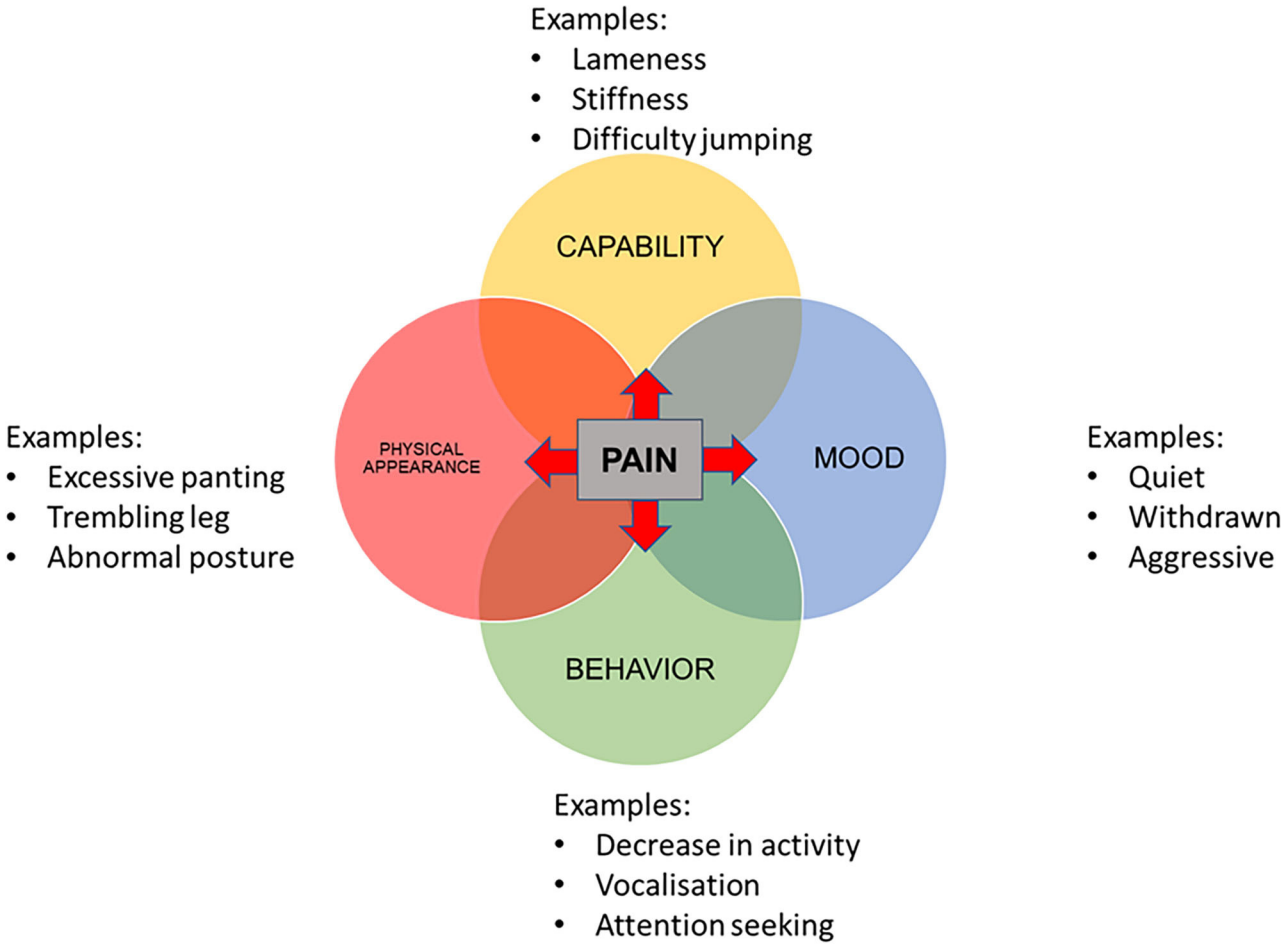
Conceptual framework for the assessment of health-related quality of life in dogs with osteoarthritis, including three example items in each domain.

The four domains were incorporated into a visual conceptual framework model (Figure 2). All four categories were deemed to be influenced by pain from osteoarthritis. Hypothesized interactions between the domains are displayed in Table 2.

**Table 2:** Hypothesised direction of interactions between the four domains represented in the conceptual framework for HRQOL in dogs with osteoarthritis (Figure 2).

	**Impact on**			
	**Physical appearance**	**Capability**	**Behavior**	**Mood**
Impact by				
Physical appearance	-	No	No	No
Capability	Yes	-	Yes	Yes
Behavior	Yes	Yes	-	Yes
Mood	Yes	Yes	Yes	-

## Discussion

The current study aimed to construct a conceptual framework of indicators of HRQOL in dogs with OA, through a systematic literature review and expert panel, focusing on the subjective experience of the dog. This approach is consistent with FDA guidance (26) for the development of PROMs. The literature search revealed 21 quality, peer-reviewed studies relating to canine OA and its impact on QOL.

Over half of the studies described the development of one of five existing instruments or their translation into other languages. These instruments are varied in their relation to HRQOL in dogs with osteoarthritis. The LOAD (31) and HCPI (16, 42) instruments both focus on orthopedic pain and its impact on exercise and mobility rather than HRQOL, although the HCPI does incorporate one question each on mood and vocalization. The CBPI (15) and COI (36, 37) were also developed with the aim of assessing the impact of pain and also focus on mobility. The CBPI includes a question relating to enjoyment of life and both incorporate a global impression of QOL. However, neither include items relating to physical appearance or sociability of the dog and the COI does not refer to mood. The current study focused on measuring the experiences of dogs with osteoarthritis relating to their day-to-day life, rather than clinical signs of OA and severity of pain, allowing a more global HRQOL measurement than these earlier instruments.

More relevant to HRQOL was the Glasgow University health-related dog behavior questionnaire (GUVQuest) which was specifically developed to assess the impact of pain, including from OA, on HRQOL (8, 45, 48). This instrument appears comprehensive and includes 109 descriptor items that reflect subjective experience, each with an associated 7-point Likert-type rating scale on which responses are framed on an agree-disagree continuum. Factor analysis revealed a range of 12 purported HRQOL domains (8, 45, 48). However, the instrument is time-consuming to complete and has since been shortened to a 46-item measure of generic (rather than OA-specific) HRQOL (45).

Three studies used one of these instruments, with one performing a welfare assessment of a population of dogs (33). A further study used the HCPI to assess the use of mechanical thresholds in joints of dogs (41), and the other tested whether medication biased the response of owners when assessing the pain of their dogs with OA (38). This demonstrates the range of these clinical instruments but indicates a low level of use, although it is possible that further use of these instruments has not been reported. Similarly, there are few published studies that have used other canine QOL assessment tools (19).

Of the remaining six studies, two reported clinical-based methods to OA assessment: mechanical joint threshold (43) and thermal sensory testing (47). Although perhaps relevant to the diagnosis and monitoring of OA, these are not owner-reported measures and therefore provided no useful items to the CF in the current study. One study used clinical records to retrospectively investigate aggressive behaviors in dogs with osteoarthritis (34). This focus on behavior provided useful additions to the conceptual framework that the studies using the existing instruments, with their focus on mobility and exercise, may not have revealed. The final two studies used qualitative methods, interviewing owners of osteoarthritic dogs on their experiences (14, 35). These also provided useful items that may have been missed by the quantitative studies, such as dogs having slower and shorter walks (14).

More than half of the items were unique to a single manuscript. This was especially the case in the original domains of “energy” and “temperament” and perhaps reflects the subjectivity of these domains. There was scope for different semantic interpretations for several items because their exact meaning within the context of their respective source articles was not always clearly characterized. Combination of synonyms reduced the number of items from 134 to 47. The mobility domain had fewer unique items and contained most of the most commonly reported items. There are several potential reasons for this. It could indeed be that these are the most common issues in OA in dogs, either as the most important to owners, or the most obvious signs. This is reflected in the clinical signs reported to be useful in a presumptive OA diagnosis (49). However, aspects of mobility were strongly represented in the existing instruments, which were developed or used in nearly three quarters of the studies screened in this review.

The final conceptual framework comprised four domains representing HRQOL of dogs with osteoarthritis. The final domains were mobility, behavior, mood, and physical appearance which were all deemed to be affected by pain. A negative impact of pain on QOL has been reported in humans (50–52). It is likely that this is similar in dogs; chronic pain has a wide range of impacts including loss or difficulty in expression of normal behaviors, such as using stairs and the development of new behaviors (53, 54). Other domains were hypothesized to all impact on each other, apart from physical appearance which was impacted on by capability, behavior, and mood but did not itself have any impact on the other domains. This is an indication of the complex nature of quality of life and its assessment.

The resulting CF is consistent with a non-peer reviewed model produced by Canine Arthritis Management (CAM) representing the impact of chronic pain on capability, behavior, muscular changes and posture (55). Although a founder of CAM was present at the workshop in this study, the similarity of the models was noticed after the majority of construction of the CF was completed. Perhaps the main difference is the presence of the “mood” domain in the CF. It is likely that mood is included under the domain “behavior” in the CAM model. Results are also consistent with recent reports on a conceptual framework of HRQOL in cats with osteoarthritis using a similar methodology to the current study (30). The main difference was the removal of a “well-being” category in the current study. There were no items found to be included under “well-being” that could not be categorized under one of the other domains. There is not currently a clear distinction between the terms “well-being” and “quality of life” and it is likely that the terms overlap or are even synonymous (56). If this were the case, this would support this domain being incorporated into the other categories.

There were some limitations to this study, perhaps the main one being that the multiple references to existing pain and HRQOL instruments in the screened papers, which limited the usefulness of quantification of item frequency in this review. This may have resulted in an overestimation of the importance of mobility and exercise-based items. However, the discussion from the expert panel allowed the review of these items and mobility and exercise (termed “capability”) were deemed to be important. There may also have been bias at the review level by searching publications only in the English language. Again, the use of an expert panel allowed for the addition of any items that may have been missed in the review and no items were added.

The conceptual framework developed by this study has highlighted the complexity of HRQOL in dogs with osteoarthritis, and the impact of pain on all other HRQOL domains. It can be used as a first step in the development of a disease-specific instrument to measure HRQOL in dogs with osteoarthritis, in order to encourage and better monitor treatment. A future qualitative concept elicitation study with key informants including veterinarians and dog owners would provide additional evidence to validate whether the HRQOL domains and their interrelations in our model reflect real-world experiences.

### Resource Identification Initiative

Europe PubMed Central, RRID:SCR_005901

## Data Availability Statement

The original contributions presented in the study are included in the article/Supplementary Material, further inquiries can be directed to the corresponding authors.

## Author Contributions

AC, DB, and IO contributed to conception and design of the study. BA performed the literature search. BA, GC, SM, and LG performed sifting, data extraction, and quality assessment. Data extraction was audited by CR. AC, BA, CR, and DB planned and attended the expert workshop. ZB and HC provided expert advice at the workshop. CR wrote the first draft of the manuscript. BA wrote sections of the manuscript. All authors contributed to manuscript revision, read, and approved the submitted version.

## Funding

The authors declare that this study received funding from Zoetis under the auspices of a wider collaboration: the Veterinary Health Innovation Engine (vHive). In addition to the funding described above (Conflicts of Interest), the funder had the following involvement in the study: compensation to expert panel.

## Conflict of Interest

DB and IO are employees of company Zoetis. GC, BA, and CR's positions are funded by Zoetis, LG has a PhD scholarship which is partially funded by Zoetis. AC is an academic Principal Investigator for other projects funded or co-funded by Zoetis. The attendance of ZB and HC at the expert panel was compensated by Zoetis. The remaining author declares that the research was conducted in the absence of any commercial or financial relationships that could be construed as a potential conflict of interest.

## Publisher's Note

All claims expressed in this article are solely those of the authors and do not necessarily represent those of their affiliated organizations, or those of the publisher, the editors and the reviewers. Any product that may be evaluated in this article, or claim that may be made by its manufacturer, is not guaranteed or endorsed by the publisher.
