# Supplementary material for: Construction of a Conceptual Framework for Assessment of Health-Related Quality of Life in Dogs With Osteoarthritis
Source: Front Vet Sci. 2021 Sep 24;8:741864. doi: 10.3389/fvets.2021.741864 (PMC8497798; doi:10.3389/fvets.2021.741864)
Supplement: Supplementary file 1 [file Table_1.DOCX]

**Supplementary Table 1.** Quality assessment of 21studies from a systematic literature review on indicators of HRQOL in dogs with osteoarthritis. Reported using STROBE guidelines (29)

| **Reference** | **Year** | **Study rationale explained** | **Specific objectives/ hypothesis** | **Location/ study period** | **Eligibility criteria** | **Sample size calculation/ sample selection** | **Outcomes/ exposures/ variables described** | **Data sources described** | **Potential bias addressed** | **Statistical methods described** | **Key results summarised** | **Study limitations discussed** | **Funding/ conflict of interest described** | **Total score** |
| --- | --- | --- | --- | --- | --- | --- | --- | --- | --- | --- | --- | --- | --- | --- |
| Andersson & Bergstrom (32) | 2019 | Y | Y | Y | Y | N | Y | Y | Y | Y | Y | Y | Y | 11 |
| Baltzer et al (33) | 2019 | Y | Y | N | Y | N | Y | Y | Y | Y | Y | Y | Y | 10 |
| Barcelos et al (34) | 2015 | Y | Y | Y | Y | N | Y | Y | Y | N/A | Y | Y | N | 9 |
| Belshaw et al (35) | 2020 | Y | Y | Y | Y | Y | Y | Y | Y | N/A | Y | Y | Y | 11 |
| Belshaw et al (14) | 2020 | Y | Y | Y | Y | Y | Y | Y | Y | N/A | Y | Y | Y | 11 |
| Brown, DC (36) | 2014 | Y | Y | Y | Y | Y | Y | Y | Y | Y | Y | Y | Y | 12 |
| Brown DC.(37) | 2014 | Y | Y | Y | Y | Y | Y | Y | Y | Y | Y | Y | Y | 12 |
| Brown et al (15) | 2007 | Y | Y | N | Y | Y | Y | Y | Y | Y | Y | Y | Y | 11 |
| Essner et al (39) | 2017 | Y | Y | Y | Y | Y | Y | Y | Y | Y | Y | Y | Y | 12 |
| Essner et al (38) | 2020 | Y | Y | Y | Y | Y | Y | Y | Y | Y | Y | Y | Y | 12 |
| Harris et al (41) | 2018 | Y | Y | Y | Y | Y | Y | Y | Y | Y | Y | Y | Y | 12 |
| Hercock et al (31) | 2009 | N | Y | Y | N | N | Y | Y | Y | Y | Y | Y | Y | 9 |
| Hielm-Bjorkman et al (42) | 2003 | Y | Y | Y | Y | Y | Y | Y | Y | Y | Y | Y | Y | 12 |
| Hielm-Björkman et al (16) | 2009 | Y | Y | Y | Y | Y | Y | Y | Y | Y | Y | Y | N | 11 |
| Knazovicky et al (43) | 2016 | Y | Y | Y | Y | Y | Y | Y | Y | Y | Y | Y | Y | 12 |
| Ragetly et al (44) | 2019 | Y | Y | Y | Y | Y | Y | Y | Y | Y | Y | N | Y | 11 |
| Reid et al (45) | 2013 | Y | Y | Y | Y | Y | Y | Y | N | Y | Y | N | Y | 10 |
| Walton et al (46) | 2013 | Y | Y | Y | N | Y | Y | Y | Y | Y | Y | Y | Y | 11 |
| Williams et al (47) | 2014 | N | Y | Y | N | Y | Y | Y | Y | Y | Y | Y | Y | 10 |
| Wiseman-Orr et al (48) | 2004 | Y | Y | Y | Y | Y | Y | Y | Y | Y | Y | Y | Y | 12 |
| Wiseman-Orr et al (8) | 2006 | Y | Y | Y | Y | Y | Y | Y | Y | Y | Y | Y | Y | 12 |

**Supplementary Table 2** Original domains and items resulting from a literature review on indicators of HRQOL in dogs with osteoarthritis and their frequency of occurrence.

| **Physical appearance (n)** | **Mobility (n)** | **Energy/ vitality/ behavior (n)** | **Temperament (n)** | **Pain expressions (n)** | **Sociability (n)** |
| --- | --- | --- | --- | --- | --- |
| Panting* (3) | Rising from lying* (9) | General activity (6) | Mood* (5) | Vocalisation* (9) | Increased frequency of contact with human family (2) |
| Leg trembling or shaking* (1) | Climbing up* (9) | Willingness to play* (5) | Aggression shown when approached by people (3) | Pain at worst (3) | Comfort seeking* (2) |
| Can "see it in their eyes" * (1) | Climbing down* (8) | Resting during exercise (3) | Less enjoyment of life (3) | Pain at least (3) | Attention seeking* (2) |
| Alteration in gait* (1) | Jumping up* (7) | Change in appetite* (3) | Depressed* (3) | Sore (3) | Clingy (1) |
| Looking awkward* (1) | Jumping down* (7) | Shorter walks (2) | Unresponsive* (2) | Current pain (2) | Not following the owner around the house (1) |
| Weight gain or loss (1) | Stiffness after lying down* (4) | Slower walks (2) | Confused* (2) | Average pain (2) | Unsociable* (1) |
|  | Ease in lying down* (4) | Listless (2) | Nervous (2) | Pacing, restless, unsettled (2) | Withdrawn* (1) |
|  | Stiffness* (4) | Reluctant* (2) | Decreased confidence* (2) | Stoic (2) |  |
|  | Willingness to walk* (4) | Dog “watching as opposed to participating” in activities (1) | Sad (2) | Licking lips (1) |  |
|  | Willingness to trot* (4) | “Just not wanting to move” (1) | Aggression to other dogs (2) | Licking (1) |  |
|  | Severity of limp * after mild activities (3) | Not being able to make it home (1) | Sorrowful (2) | Accepting (1) |  |
|  | Ability to walk (3) | Not pulling on the leash anymore during walks.* (1) | Aggressive* (2) |  |  |
|  | Ability to run (3) | Hiding (1) | Inconsistent (2) |  |  |
|  | Difficulty in movement after heavy exercise (3)) | Frequency of tail wag (1) | Submissive (1) |  |  |
|  | Willingness to gallop* (3) | More time resting (1)* | Inconsistent (1) |  |  |
|  | Willingness to exercise (2) | Differences in fitness (1) | Pathetic (1) |  |  |
|  | Severity of stiffness on waking (2) | Differences in athleticism (1) | Pitiful (1) |  |  |
|  | Difficulty with joint (2) | Difference in relaxed state (1) | Miserable (1) |  |  |
|  | Stiffness during the day* (2) | Energy (1) | Uncomfortable (1) |  |  |
|  | “Paying for it” after chasing (2) | Apathetic (1) | Agitated (1) |  |  |
|  | Increased pain or stiffness the following day* (2) | Apprehensive* (1) | Disturbed (1) |  |  |
|  | Severity of limp after moderate activities (2) | Lackluster (1) | Miserable (1) |  |  |
|  | Limping after moderate activities* (2) | Lethargic* (1) | Negative temperament (1) |  |  |
|  | Limping* (2) | More time sniffing* (1) | Bites directed at extremities (1) |  |  |
|  | Staying upright in a moving car* (1) | Reluctant to go over rough terrain (1) | Resigned (1) |  |  |
|  | Posturing to toilet* (1) | Sleepy (1) | Unhappy (1) |  |  |
|  | Effect of cold and damp weather on ability to exercise (1) | Slowed (1) | Aggression shown when recumbent dog approached (1) |  |  |
|  | Difficulty maintaining a 'hup' position (1) | Sluggish (1) | Grumpy (1) |  |  |
|  |  | Tired (1) | Irritable* (1) |  |  |
|  |  | Weary (1) | Territorial (1) |  |  |
|  |  | Detached* (1) | Protective* (1) |  |  |
|  |  | Quiet* (1) | Anxious (1) |  |  |
|  |  | Keen(1) | Cautious (1) |  |  |
|  |  | Compulsive (1) | Distressed (1) |  |  |
|  |  | Eager (1) | Frightened* (1) |  |  |
|  |  |  | Aggression: short and easy to break up incidents (1) |  |  |
|  |  |  | Panicky (1) |  |  |
|  |  |  | Strained (1) |  |  |
|  |  |  | Uneasy (1) |  |  |
|  |  |  | Upset (1) |  |  |
|  |  |  | Dull (1) |  |  |
|  |  |  | Aggression: wide range of targets (1) |  |  |
|  |  |  | Uninterested* (1) |  |  |
|  |  |  | Dogs feeling “vulnerable” or “needy” (1) |  |  |
|  |  |  | At ease (1) |  |  |
|  |  |  | Panicky (1) |  |  |
|  |  |  | Concerns about the dog’s “happiness” (1) |  |  |

*items that were presented to the attendees of the expert panel

**Supplementary Table 3.**  Study characteristics of 21 studies from a systematic literature review on indicators of HRQOL in dogs with osteoarthritis.

| \| **Reference** \| **Study design** \| **Population (n)** \| **Intervention** \| **Comparison (n)** \| **Outcomes/ purpose of study** \| \| --- \| --- \| --- \| --- \| --- \| --- \| \| Andersson & Bergstrom (32) \| Cross sectional, \| Dogs with elbow osteoarthritis (OA) (117) \| Canine Orthopaedic Index (COI) \| Healthy dogs (146) \| Validation of COI in Swedish \| \| Baltzer et al (33) \| Observational, prospective \| Police dogs (158) \| COI and functional assessment of fitness (FA) \| N/A \| COI and FA scores \| \| Barcelos et al (34) \| Retrospective qualitative \| Aggressive dogs with musculoskeletal pain (11) \| N/A \| Healthy aggressive dogs (11) \| Preliminary comparison of aggressive dogs \| \| Belshaw et al (35) \| Qualitative \| Owners of osteoarthritic dogs (35) \| N/A \| N/A \| Impact on the owner \| \| Belshaw et al (14) \| Qualitative \| Owners of osteoarthritic dogs (35) \| N/A \| N/A \| Changes in dog walks \| \| Brown (36) \| Qualitative \| Owners of osteoarthritic dogs (87) \| N/A \| N/A \| Develop of COI questions \| \| Brown (37) \| Cross sectional \| Owners of osteoarthritic dogs (100) \| COI \| N/A \| Psychometric testing of COI \| \| Brown et al (15) \| a) qualitative  b) cross sectional \| Owners of dogs with osteoarthritis (a) 36; b) 70) \| a) N/A  b) Canine Brief Pain Inventory (CBPI) \| a) N/A  b) Owners of healthy dogs (50) \| a) Development of CBPI  b) psychometric testing of CBPI \| \| Essner et al (39) \| Observational, cross sectional \| Owners of dogs with osteoarthritis (58) \| CBPI \| Owners of healthy dogs (21) \| Evaluation of CBPI in Swedish \| \| Essner et al (38) \| Cross sectional \| Dogs with OA (71) \| CBPI, Helsinki Chronic Pain Index (HCPI) \| N/A \| Risk factors for chronic pain and probability of response bias \| \| Harris et al (41) \| Cross sectional \| Dogs with hindlimb OA (27) \| HCPI and mechanical thresholds \| Healthy controls (28) \| Use of mechanical thresholds \| \| Hercock et al (31) \| Prospective Cohort \| Dogs with elbow OA (26) \| LOAD tool \| N/A \| Validation of LOAD tool \| \| Hielm-Bjorkman et al (42) \| Prospective cross sectional \| Owners of dogs with hip dysplasia (41) \| Pain questionnaire \| Owners of healthy dogs (24) \| Assessment of pain questionnaire \| \| Hielm-Björkman et al (16) \| a) cross sectional  b) cohort \| a) Dogs with OA (61)  b) Dogs with OA receiving carprofen (17) \| a) HCPI  b) HCPI \| a) N/A  b) Dogs with OA receiving placebo (17) \| a) Psychometric testing of HCPI  b) sensitivity of HCPI \| \| Knazovicky et al (43) \| Cross sectional \| Dogs with hip/stifle OA (31) \| Thermal quantitatitve sensory testing (QST) \| Normal dogs (23) \| Use of canine OA as a model for human OA \| \| Ragetly et al (44) \| Prospective clinical \| Dogs with OA (32) \| CBPI French version \| N/A \| Validation of CBPI in French \| \| Reid et al (45) \| Cross sectional \| Volunteers from the department of statistics (8)  b)Unwell dogs (90) \| a)N/A  b)Glasgow University Veterinary School Questionnaire (GUVQuest) short form \| a) N/A  b)Normal dogs (35) \| a) Development of GUVQuest  b) Validation of GUVQuest \| \| Walton et al (46) \| Prospective cross sectional \| Dogs with OA (222) \| LOAD, HCPI, CBPI \| N/A \| Validation of LOAD tool \| \| Williams et al (47) \| Prospective observational \| Dogs with OA (9) \| Thermal quantitatitve sensory testing (QST) \| Healthy dogs (23) \| Usability of QST in dogs \| \| Wiseman-Orr et al (48) \| a) Qualitative  b) Cross sectional \| a) Owners of dogs with OA (17)  b) Owners of healthy dogs (165) \| a) N/A  b) HRQOL questionnaire \| N/A \| a) development of HRQOL questionnaire  b) primary validation of HRQOL questionnaire \| \| Wiseman-Orr et al (8) \| Cross sectional \| Dogs with chronic degenerative joint disease (108) \| GUVQuest \| Healthy dogs (26) \| Validation of GUVQuest \| |  |  |
| --- | --- | --- | --- | --- | --- | --- | --- | --- | --- | --- | --- | --- | --- | --- | --- | --- | --- | --- | --- | --- | --- | --- | --- | --- | --- | --- | --- | --- | --- | --- | --- | --- | --- | --- | --- | --- | --- | --- | --- | --- | --- | --- | --- | --- | --- | --- | --- | --- | --- | --- | --- | --- | --- | --- | --- | --- | --- | --- | --- | --- | --- | --- | --- | --- | --- | --- | --- | --- | --- | --- | --- | --- | --- | --- | --- | --- | --- | --- | --- | --- | --- | --- | --- | --- | --- | --- | --- | --- | --- | --- | --- | --- | --- | --- | --- | --- | --- | --- | --- | --- | --- | --- | --- | --- | --- | --- | --- | --- | --- | --- | --- | --- | --- | --- | --- | --- | --- | --- | --- | --- | --- | --- | --- | --- | --- | --- | --- | --- | --- | --- | --- | --- | --- | --- |
